# Incomplete Susac Syndrome With Recurrent Retinal Occlusions and Vertigo: A Case of Early Immunosuppressive Success

**DOI:** 10.7759/cureus.90088

**Published:** 2025-08-14

**Authors:** Arathi Kulkarni, Amara Miller, Chandana Keshavamurthy, Tamim Sultani, Ramin Schadlu, Trent Smith

**Affiliations:** 1 Internal Medicine, University of Arizona College of Medicine - Phoenix, Phoenix, USA; 2 Rheumatology, University of Arizona College of Medicine - Phoenix, Phoenix, USA; 3 Radiology, University of Arizona College of Medicine - Phoenix, Phoenix, USA; 4 Ophthalmology, University of Arizona College of Medicine - Phoenix, Phoenix, USA

**Keywords:** adult rheumatology, branch retinal artery occlusion, central retinal artery occlusions, susac syndrome, vertigo diagnosis

## Abstract

Susac syndrome (SS) is a rare autoimmune microangiopathy that targets the brain, retina, and inner ear. Its variable and often incomplete clinical presentation frequently leads to misdiagnosis, increasing the risk of morbidity. We report the case of a 53-year-old Caucasian woman with a history of pityriasis lichenoides et varioliformis acuta (PLEVA) and severe myopia. She experienced recurrent branch retinal artery occlusion (BRAO) and retinal vein occlusion, following an episode of intense vertigo. Notably, her initial vascular occlusion occurred prior to the onset of vertigo. A comprehensive diagnostic evaluation excluded other potential etiologies, including systemic lupus erythematosus (SLE), Takayasu arteritis, giant cell arteritis (GCA), and other forms of vasculitis. Her clinical course, characterized by repeated retinal vascular events and episodes of peripheral vertigo, supported a diagnosis of incomplete SS. Incomplete SS is a phenotype in which two organ systems, in this case the retina and inner ear, are affected and events may be asynchronous. Early initiation of immunosuppressive therapy, including corticosteroids and mycophenolate mofetil, led to significant improvement in her vision. This case highlights the critical importance of early recognition and treatment of incomplete SS to prevent irreversible organ damage.

## Introduction

Susac syndrome (SS) is a rare immune-mediated, pauci inflammatory, ischemia-causing occlusive microangiopathy that affects the brain, retina, and inner ear microvasculature. It was first described in patients who presented with personality changes and associated with multiple branch retinal artery occlusions (BRAO) [1]. This condition primarily affects young Caucasian adults, with a 3:1 greater prevalence in women than men [2]. The age of onset typically ranges from 16 to 40 years [1]. The incidence rate is unknown, with only a few hundred cases published in the literature [2].

Medications, including interferon beta treatment and checkpoint inhibitors, and infections such as COVID-19 have been found as possible triggers for the syndrome [1]. Pathogenesis is unknown, but a widely accepted hypothesis suggests that cytotoxic CD8+ T lymphocytes, activated due to an unknown antigen, can cause enhanced endothelial permeability of the blood-brain barrier [3], resulting in an increase in CD8+ T lymphocytes in both blood and cerebrospinal fluid (CSF). Other proposed mechanisms, such as the vessel wall leakage phenomenon, have also been suggested [2]. Although antibody testing is not routinely done, Susac et al. proposed in 2007 that anti-endothelial cell antibodies (AECA) play a role in the pathogenesis of SS, supporting the autoimmune basis for this condition [4].

Although not always fully observed, the complete triad associated with this condition includes central nervous system (CNS) dysfunction with encephalopathy, retinal artery branch vaso-occlusions, and sensorineural hearing loss [5]. The diagnostic criteria proposed by the European Susac Consortium offer guidance for diagnostic evaluation, categorizing cases into definite, probable, and possible levels of suspicion [5]. A definitive diagnosis is when all three criteria are met, and each organ system is affected. Probable diagnosis is defined by two or three systems being affected. Finally, a possible SS diagnosis is considered when a patient shows the clinical or paraclinical findings of the triad but does not fulfill the criteria. In a review of 304 published cases of SS from 2013, it was reported that only 13% of patients met the clinical triad entities at disease onset, which commonly leads to misdiagnosis [6]. Common mimics of SS include multiple sclerosis (MS), Meniere disease, cerebral vasculitis, stroke, histoplasmosis, viral encephalitis, Hashimoto’s encephalitis, and CNS lymphoma [6]. An entity known as incomplete SS has also been reported when only two of the three organ symptoms have been found to be affected instead of the complete triad [7].

Retinal involvement in SS can manifest as photopsia, black spots, scintillating scotomas, and painless visual loss [8,9]. In addition, retinal damage can occur peripherally without affecting overall function and encephalopathy may render the patient unaware of any deficits in their vision [9]. Fluorescein angiography can be used to detect BRAOs indicating retinal branch ischemia.

Sensorineural hearing loss can occur in up to 90% of patients, which can be abrupt and bilateral in SS [10]. However, most common auditory dysfunction is tinnitus and ear fullness. A pure-tone audiogram shows hearing loss in the low to mid-tone range due to ischemic damage to the apical part of the cochlea [1,8,11]. Rotatory vertigo due to vestibular damage has also been reported.

CNS manifestations can be mild, moderate, or severe, depending on the degree of ischemia in SS. Headaches, cognitive dysfunction, confusion, and behavioral and personality change along with focal neurologic symptoms have all been reported [1]. MRI brain findings can reveal "snowball" lesions involving the white matter and the corpus callosum, which are small, usually round, T2 hyperintense lesions best seen on FLAIR sequences, which may or may not demonstrate enhancement. Noninvasive procedures like MRI or retinal angiography have sufficiently have higher diagnostic yield warranting brain biopsy unnecessary. Pathologic findings to note in the brain include endothelial damage in the precapillary arterioles, ranging from mummification to frank necrosis of endothelial cells [4].

Early diagnosis and treatment are crucial to managing symptoms and preventing long-term complications. Organs involved can easily be irreversibly damaged, and the window of protection is narrow. It is important to note that there are no biomarkers for disease activity. The disease can present in acute forms such as monophasic, self-limiting, and fluctuating or in polycyclic or chronic forms [12].

## Case presentation

**Figure 1:**
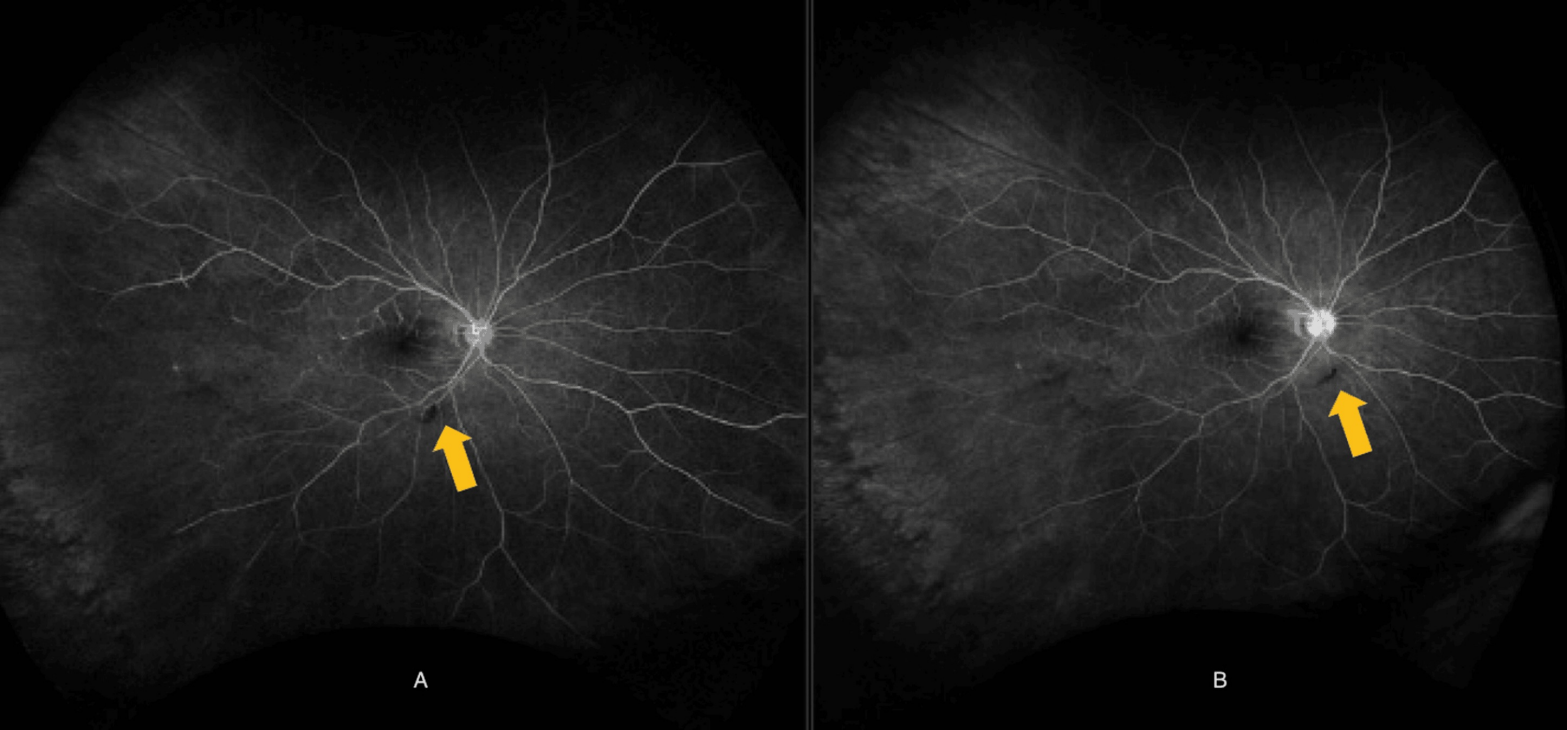
Formation of branch retinal artery occlusions (BRAOs) after symptom onset via fluorescein angiography. Arrows pointing toward BRAO. Image A (left side) is the oculus dexter (OD) and image B (right side) is the oculus sinister (OS).

**Figure 2:**
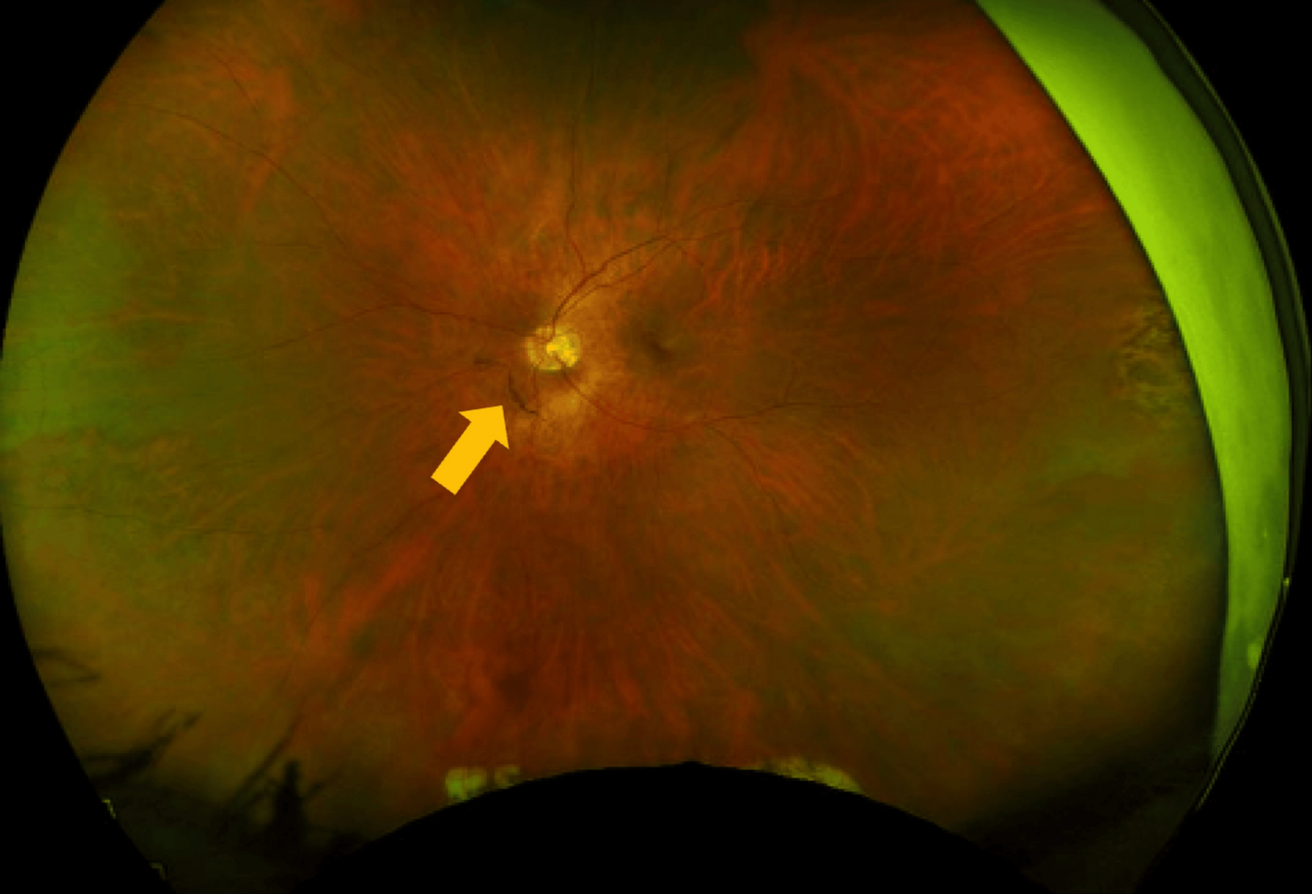
Formation or branch retinal artery occlusion (BRAO) in the fundus after symptom onset. Arrow pointing toward BRAO. The image above is the oculus sinister (OS).

A 53-year-old Caucasian female with a significant medical history of pityriasis lichenoides et varioliformis acuta (PLEVA) and cerebral palsy had been on methotrexate 12.5 mg weekly and folic acid 1 mg daily for several years. She had severe myopia and a history of retinal tears. Approximately one year prior to rheumatology evaluation, she experienced BRAO and branch retinal vein occlusion (BRVO) in her right eye. Unfortunately, an extensive autoimmune panel or a search for underlying causes of this event was not conducted at that time. The patient did not recall receiving treatment, and since her vision was not significantly compromised, she did not seek further evaluations or investigations. A few months later, she was hospitalized for refractory rotatory vertigo. Cardiac evaluations, including telemetry monitoring and an echocardiogram, were unremarkable, and a neurologic workup ruled out acute stroke, meningitis, and encephalitis. An MRI of the brain was unremarkable, and an ENT evaluation was not performed at that time. Imaging studies were initially conducted to evaluate her vertigo further. A CT angiography (CTA) of the neck, chest, and head showed no abnormalities. This brief episode then resolved without significant complications, although she reported intermittent residual dizziness for several months following resolution of the peripheral vertigo. A few months later, the patient began to report cloudiness in her right eye and objectively diminished vision. Fluorescein angiography revealed inflammation in the arterial wall and the presence of BRAO and BRVO. Color fundus photographs and optical coherence tomography (OCT) images indicative of BRAO were obtained. Key observations included that the retina distal to the occlusion site appeared white, with marked inner retinal hyperreflectivity. Figure 1 and Figure 2 show the patient's condition with the initial onset of BRAOs via fluorscein angiography and fundus imaging.

We investigated the possible etiologies for BRAOs and central retinal artery occlusions (CRAO), exploring carotid and thromboembolic etiologies. A thorough workup of other ischemic, toxic, and metabolic insults yielded negative results.

A follow-up MRI of the brain was performed and compared to an earlier scan. The results showed small areas of encephalomalacia and gliosis affecting the left posterolateral putamen, the lateral margin of the left caudate head, regions within the left basal ganglia, and partial involvement of the posterior limb of the left internal capsule. These findings suggest a chronic ischemic insult in the left hemisphere, possibly related to a prior ischemic event. Notably, these abnormalities were absent on the MRI conducted during the evaluation for vertigo. The patient denied experiencing any new neurological symptoms when the MRI was repeated. She reported no headaches, delirium, seizures, or psychosis. We were unable to determine when the ischemic event occurred. The absence of risk factors such as diabetes, hypertension, and hyperlipidemia raised concerns about other potential underlying causes. Although we did not observe snowball lesions in the white matter or central callosal fibers of the corpus callosum, there are cases in which small round T2 hyperintense lesions in the basal ganglia and thalamus can be seen on FLAIR sequences with or without enhancement, in SS. This possibility increased our clinical suspicion.

The patient’s skin rashes on her trunk and extremities were attributed to PLEVA. She mentioned having oral ulcers approximately four times a year; however, these appeared to be consistent with mucositis rather than the ulcers typically associated with Behçet's disease. The patient also reported rare occurrences of vaginal ulcers but did not meet the classification criteria for Behçet's disease.

There was no history of deep vein thromboses (DVTs) or pulmonary aneurysms. She denied any history of folliculitis, hair loss or alopecia, pathergy reactions during blood draws, or photosensitive rashes. Although she mentioned experiencing sicca symptoms, she did not present any symptoms indicative of Sjögren's disease. In addition, she had arthralgias without any signs of inflammatory arthritis.

Systemic lupus erythematosus (SLE) diagnosis was ruled out due to the absence of relevant symptoms and negative results for antinuclear antibodies (ANA), double-stranded DNA tests, and Smith antibodies. To evaluate the presence of aneurysms and other vascular pathology related to vasculitis, a computed tomography angiography and magnetic resonance angiography of the neck, chest, abdomen, and pelvis were performed, all of which returned negative results. Furthermore, the patient did not display cutaneous, pulmonary, or renal manifestations typical of small vessel vasculitis and tested negative for myeloperoxidase (MPO) and proteinase 3 (PR3) antibodies.

Routine laboratory evaluations indicated mild anemia, while white blood cell and platelet counts remained within normal limits. Both prothrombin time (PT) and partial thromboplastin time (PTT) were within acceptable ranges. Inflammatory marker levels were also within the normal range. Urinalysis did not show any presence of protein or blood, and urine toxicology screenings returned negative results for illicit substances. In addition, the patient tested negative for cryoglobulins. Monoclonal proteins were not detected in serum protein electrophoresis and immune fixation electrophoresis. Infectious disease screenings, including tests for tuberculosis, syphilis, and HIV, were all negative. Hormonal evaluations, thyroid function assessments, and vitamin D levels were all within normal parameters. Tests for thrombophilia, hyperviscosity, hypercoagulability, the antiphospholipid antibody panel, and inflammation were unremarkable. The cardiology team conducted a workup to check for potential emboli, including a transthoracic echocardiogram (TTE), which was unremarkable. There was no evidence of infective endocarditis.

A lumbar puncture, primary angiogram, and MRI of the entire spine were all normal. CSF analysis did not yield any inflammatory fluid. The vestibulocochlear evaluation and audiogram returned to normal results; however, these tests were conducted many months after her vertigo had resolved.

**Figure 3:**
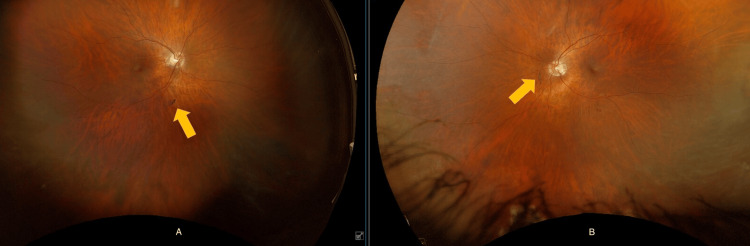
Dramatic improvement in retino-vascular findings along with resolving branch retinal artery occlusions (BRAOs) six months after immunosuppressive treatment. Image A (left side) is the oculus dexter (OD), and image B (right side) is the oculus sinister (OS).

The patient was started on 60 mg of prednisone daily for four weeks, with the dosage tapered over the subsequent six months. Mycophenolate was introduced as a steroid-sparing agent. Following the steroid treatment, an ophthalmology evaluation showed excellent objective improvement. Her vision improved. A repeat fluorescein angiography revealed a remarkable response; her visual acuity improved from 20/100 to 20/25. Figure 3 shows repeat fundus imaging after six months of treatment. She did not have any more episodes of vertigo and no new neurologic deficits. Aspirin and statins were also initiated for the latter's anti-inflammatory benefits. 

A diagnosis of incomplete SS was eventually made. This diagnosis was based on the two episodes of BRAOs, the acute onset of vertigo prior to the recurrence of ocular symptoms, and the findings of ischemic damage on the brain MRI. This was determined in an otherwise healthy female after an extensive evaluation that eliminated all potential causes.

## Discussion

Following the diagnosis of BRAOs, a comprehensive investigation was initiated into differential diagnoses including SLE, Takayasu arteritis, giant cell arteritis (GCA), and vasculitis.

The diagnosis of SLE was excluded based on negative antibody results for antinuclear antibodies (ANA), double-stranded DNA tests, smith antibodies, and the absence of lupus-related symptoms, except for oral ulcers. Systemic lupus ocular manifestations include uveitis and lupus retinopathy with extensive arteriolar occlusions and bilateral macular infarctions which did not exist in this patient [13]. We investigated the possible etiologies for BRAOs and central retinal artery occlusions (CRAO) and the sources for both conditions include carotid and thromboembolic etiologies. While small vessel disease is more often associated with BRAO, cardioembolic causes can lead to CRAO [14]. While the cause of CRVO was indeterminate in this case, high-dose steroids to treat the BRAO resolved the CRVO. Hence, we hypothesized that BRAO caused disruption of blood flow dynamics in the retina, leading to clot formation in the central vein. Managing combined vascular occlusions is controversial due to the condition's rarity. Therefore, no official guideline is currently available.

Furthermore, the patient did not conform to the demographic characteristics typically associated with GCA; thus, the retinal artery occlusions were not attributed to this condition. Takayasu's arteritis and the possibility of medium vessel vasculitis, such as polyarteritis nodosa, were also considered. A CTA and MRA of the neck, chest, abdomen, and pelvis were ordered to assess for aneurysms and other vascular pathology associated with vasculitis, which were all negative. Moreover, the patient did not exhibit cutaneous, pulmonary, or renal manifestations characteristic of small vessel vasculitis, testing negative for both myeloperoxidase (MPO) and proteinase 3 (PR3) antibodies. Neurosarcoidosis was on the differential since retinal vasculitis is reported with the disease. However, the patient had no other symptoms of sarcoidosis. 

Notably, a missed opportunity occurred during the patient’s vertigo episode; no ENT evaluation or audiologic testing was conducted despite significant symptoms prompting hospitalization. This highlights the difficulty of diagnosing SS when it presents with incomplete or sequential features. This case also underscores the critical role of fluorescein angiography and OCT in diagnosing SS, even in patients lacking the complete triad. Timely recognition and treatment of incomplete presentations are essential to preventing long-term disability.

There is no current standard treatment paradigm for SS and no standardized scoring to determine prognosis. However, early identification and treatment of SS can prevent long-term sequela of permanent neurological, auditory, and visual loss. Current treatments include IV pulse steroids, followed by high-dose oral steroids, IVIG, rituximab, MMF, and, in some cases, plasmapheresis, which has been seen in case reports to improve patient outcomes [15]. The current literature has been varied on the doses of steroids. Studies have shown that the use of IV methylprednisolone for three days, followed by a modest course of oral daily prednisone 40 to 60 mg for two weeks, and then by a decrease of 5 to 10 mg every one to two weeks, has provided favorable outcomes [1,16]. IVIG at doses of 2 gm/kg followed by 1 g/kg every two weeks has also been recommended. However, recurrences of SS have also been reported in the proximal vessels despite being on immunosuppression [12].

Treatment depends on which component of the clinical triad poses the greatest clinical effect. In this patient’s case, it was the retinal involvement. A more aggressive, more prolonged course of treatment is recommended in patients with highly severe organ presentations such as acute visual and auditory loss and encephalopathy [12]. Given the fact that less than 20% of patients exhibit the complete clinical triad, in addition to any one of the triad components possibly being the first and only manifestation, we did not want to delay the diagnosis in this patient’s case and wait for the other components to develop particularly given the unexplained vertigo and the abnormal brain MRI findings.

## Conclusions

A diagnosis of SS was made based on two episodes of BRAO, in association with CRVO. We hypothesize that chronic ischemic changes seen on brain MRI, a single episode of severe vertigo, and the absence of cardiovascular or hypercoagulable risk factors align with a diagnosis of incomplete SS. Although these manifestations occurred non-concurrently, they all presented within a year, suggesting a shared pathophysiologic process. This case emphasizes the need for high clinical suspicion when evaluating recurrent retinal vascular events and unexplained vertigo, especially in young women, even in the absence of the full clinical triad.

Although the precise mechanism of CRVO remains uncertain in this case, it is notable that high-dose corticosteroids initiated for BRAO led to the resolution of both arterial and venous occlusions. We postulate that inflammation-induced disruption of retinal hemodynamics following BRAO may have contributed to secondary CRVO. Emerging literature supports early immunosuppressive therapy, commonly steroids, IVIG, and/or mycophenolate, in improving neurological and ophthalmologic outcomes in SS. In our case, early immunosuppression halted disease progression and facilitated complete recovery of visual function.
